# Jellyfish Stings and Their Management: A Review

**DOI:** 10.3390/md11020523

**Published:** 2013-02-22

**Authors:** Luca Cegolon, William C. Heymann, John H. Lange, Giuseppe Mastrangelo

**Affiliations:** 1 Department of Molecular Medicine, Padua University, Padua 35128, Italy; E-Mail: giuseppe.mastrangelo@unipd.it; 2 School of Public Health, Imperial College London, St. Mary’s Campus, London WC2 1PG, UK; 3 Sarasota County Health Department, Sarasota, FL 34237, USA; E-Mail: william_heymann@doh.state.fl.us; 4 Envirosafe Training and Consultants, 2366 Golden Mile Highway, Pittsburgh, PA 15239, USA; E-Mail: jhlange1@hotmail.com

**Keywords:** jellyfish, cnidarians, cubozoans, stings, envenomation, pain, evidence-based treatment, vinegar, hot water, ice pack

## Abstract

Jellyfish (cnidarians) have a worldwide distribution. Despite most being harmless, some species may cause local and also systemic reactions. Treatment of jellyfish envenomation is directed at: alleviating the local effects of venom, preventing further nematocyst discharges and controlling systemic reactions, including shock. In severe cases, the most important step is stabilizing and maintaining vital functions. With some differences between species, there seems to be evidence and consensus on oral/topical analgesics, hot water and ice packs as effective painkillers and on 30 s application of domestic vinegar (4%–6% acetic acid) to prevent further discharge of unfired nematocysts remaining on the skin. Conversely, alcohol, methylated spirits and fresh water should be carefully avoided, since they could massively discharge nematocysts; pressure immobilization bandaging should also be avoided, as laboratory studies show that it stimulates additional venom discharge from nematocysts. Most treatment approaches are presently founded on relatively weak evidence; therefore, further research (especially randomized clinical trials) is strongly recommended. Dissemination of appropriate treatment modalities should be deployed to better inform and educate those at risk. Adequate signage should be placed at beaches to notify tourists of the jellyfish risk. Swimmers in risky areas should wear protective equipment.

## 1. Introduction

**Figure 1:**
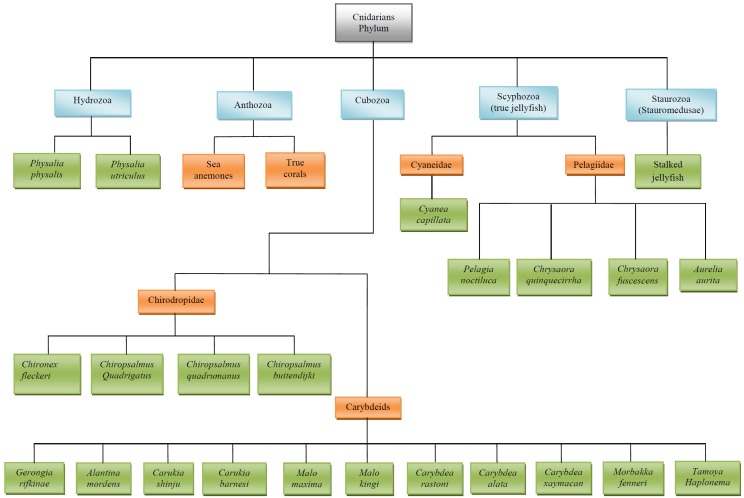
Cnidarians phylum: Main species byfamily and class.

Jellyfish belong to the phylum Cnidarians. The phylum is subdivided into five classes (see Figure 1: Staurozoa (Stauromedusae); Scyphozoa (true jellyfish); Hydrozoa (Portuguese Man O’ War, fire corals and hydroids); Cubozoa (box jellyfish); and Anthozoa (sea anemones and true corals) [1]) and is composed of about 10,000 species, with 100 of them known to be dangerous to humans [2,3].

Jellyfish have a bell-shaped body (umbrella) of different sizes, with a varying number of tentacles, depending on the species. The tentacles reach from a few millimeters up to 40 m in length, depending on the species, with their color ranging from transparent to whitish, yellowish, purple or bluish [2,3].

The tentacles present specialized epidermic cells, called cnidocytes, that contain three categories of organelles, called cnidae (and also cnidocysts). Nematocysts, one of the three categories of cnidae, are hollowed capsules containing a tightly coiled and folder thread immersed in the cnidarians’ venom [1,2,4]. Nematocyst thread tubules evert so that the venom is injected on the outside of the thread tube. Some thread tubes are also hollowed and can discharge venom through the end [1,2,3]. Tentacles contain from a few thousand to several billion nematocysts [1,2].

Nematocysts are discharged onto the skin within a fraction of a second, making a jellyfish nematocyst discharge one of the most rapid mechanical events in nature [5]. Nematocysts can function even when separated or if the organism is dead, although discharge rate decreases after death [1,6,7].

Discharge of the jellyfish venom is triggered by mechanical stimuli (such as skin rubbing or tentacle traction) [8], sudden increase in the osmotic pressure of the capsular fluid due to the removal of bound calcium ions [9] and sudden relaxation of spring-like tensions in the nematocyst collagen framework [5]. The above stimuli activate the uncoiling of the thread, which penetrates into the tissues, causing the nematocyst to discharge venom [8,10].

Although all cnidarians are capable of envenomation, most are harmless to humans, as some do not have nematocyst shafts of sufficient length to enable the thread to deposit toxins deep enough into the epidermis [1,11] or might produce toxins that do not cause significant harm to humans [12]. Harmful cnidarians include vertebrate feeders or bigger jellyfish able to release large amounts of toxin [1,2].

Jellyfish are present in all oceans of the world [2,3], with their stings being commonly observed in warm tropical marine waters [13,14], as well as in more northern regions, such as the United Kingdom [15], France [16] and Norway [17]. The geographic distribution of jellyfish seems to be undergoing an impact by global warming [18,19,20].

It has been estimated there are 150 million jellyfish stings a year [21], with some Pacific areas reporting up to 800 daily events at one single beach [22,23]. Therefore, beyond being a public health issue, jellyfish also constitute a threat to tourism [24].

Skin contact with nematocysts resembles a prick, and the subsequent inflammation and nerve irritation produces pain, swelling and itching, potentially leading to skin necrosis in more severe stings (often from Australian chirodropid cubozoans) [2,25,26]. The local effect of the venom is due to the penetration of the thread and the activity of various compounds, like phospholipase A2 [7,27], as well as exocytosis of mast cell granules (and, thus, possibly histamine release) [28,29]. Nematocysts can also cause potential systemic symptoms—as a result of the toxins entering the general circulation [1]—including gastrointestinal (mainly *Physalia physalis* and Pelagiidae spp.), muscular (*Physalia* and cubozoans spp.), cardiac (*Physalia* and cubozoans spp.), neurological (*Physalia* and cubozoans spp.) and allergic manifestations (Pelagiidae and cubozoans spp.). Jellyfish toxins reportedly also include hemolytic and lethal fractions [1,30,31]. The lethal fractions may contain cardiotoxins, able to produce ventricular arrhythmias and cardiac arrest, and neurotoxins [1,30,31], which may cause respiratory failure and respiratory arrest [32]. Intravascular hemolytic fractions can also precipitate acute renal failure [1]. Cnidarians’ venom is also immunogenic, capable of generating antibody response [1,4,24].

There has been a wealth of research done on the treatment of cnidarians’ stings, but confusion still exists as to what is the most effective first aid and clinical management [32,33,34,35]. Hence, there is an urgent need to consolidate a shared protocol for the management of these accidents.

Several authors have summarized the literature on the jellyfish phenomenon and the evidence concerning the various treatment approaches. Our aim was to integrate the knowledge reported by the most authoritative reviews and most recent research papers.

## 2. Methods

PubMed was searched for the following terms: “(jellyfish OR cnidarians OR medusae OR cubozoans OR *Physalia* OR Portuguese Man O’ War OR carybdeids or Pelagiidae OR cyanidae) AND (envenomation OR sting OR pain OR tentacles OR treatment OR management OR nematocyst OR discharge OR vinegar OR sea water OR alcohol OR ice OR cold water OR ammonia OR antidote OR methylated spirits)”, selecting only the reviews. Four of them were considered on the basis of the title, abstract, relevant and informative content, study area, profile of authors and journals. Using a function of PubMed, all the related citations were retrieved, discarding identical items and parts of publications by the same first author after examining the title and abstract. Further relevant articles were also retrieved from the referenced studies, employing Google Scholar in addition to PubMed. Reviews, commentaries, editorials, letters to editors, randomized and nonrandomized controlled trials, observational/case series studies with and without controls, laboratory studies, case reports and expert opinions were considered. Articles in English were largely preferred, and priority was given to works from the most reputable authors published in more established and referenced journals and books.

## 3. Main Stinging Pelagic Cnidaria (Figure 1) Illustrates the Taxonomy of Cnidarians

### 3.1. *Physalia* Species

*Physalia* species are improperly believed to be “jellyfish”: They are a Siphonophore colony of animals in the class hydrozoa. Two major *Physalia* spp. are commonly classified within this group [1,2]:

(1) * Physalia physalis* (the Portuguese Man O’ War): This worldwide species has a boat-like pneumatophore 2–25 cm long, with multi-tentacles measuring from 10 m up to 30 m [1]; yet, it is not a true jellyfish. This species is responsible for a large number of stings, with some being fatal.
(2) * Physalia utriculus* (the Blue Bottle): This Indo-Pacific, Indian Ocean and South Atlantic species is smaller than *P. physalis*, the float being up to 10 cm long and 5–6 cm wide, with a predominant fishing tentacle extending a maximum of 2–5 m [1,36].

The pneumatophore and the predominant tentacle are usually bright bluish, although in the Atlantic specimens, they may be purplish. This float allows them to sit on the surface of the ocean and lean horizontally when the wind blows, sailing with the wind, as well as being carried by water currents [2,3,26].

*Physalia* spp., which are responsible for a substantial proportion of cnidarians’ stings worldwide, have a widespread distribution [37]. Both *Physalia physalis* and *Physalia utriculus* may be found in Indo-Pacific areas, the Indian Ocean and the South Atlantic [1], whereas in the North Atlantic and European coasts, only the Portuguese Man O’ War has been reported [38]. *Physalia* spp. are more frequently found in hot and temperate waters [2,16,26]; however, these hydrozoans can occasionally be found cast ashore in cold Atlantic waters (e.g., North France, Belgium and the South West of England). In Australia, another multi-tentacled *Physalia* sp. has been reported [1].

As they float on the ocean surface, often in big swarms, *Physalia* spp. represent a potentially significant hazard to swimmers. *Physalia* stranded on beaches are still capable of stinging if handled, even after several days of dehydration [1].

*Physalia* stings are normally quite painful and severe, with the Portuguese Man O’ War potentially also causing major systemic symptoms [1]. *Physalia* stings are normally multiple, always causing local symptoms with instantaneous pain: linear, crossed skin wheals longer than 20 cm with intradermal oedema in the areas having contact with the tentacles [1]. Some severe skin injuries become necrotic within 24 h, evolving into scabs lasting for about two weeks before resolving and causing enduring (if not permanent) erythematous scars [16]. The skin lesions of *Physalia* are quite easy to recognize, with typical spherical nematocysts detected by skin scrapings [1].

*Physalia utriculus* usually causes local pain only and, very rarely, minor systemic symptoms. By contrast, many patients with *Physalia physalis* stings suffer systemic symptoms, which generally are gastrointestinal (abdominal ache and nausea/vomiting) and/or muscular (spasms, pain). However, severe envenomations from *Physalia* may even produce neurological (headache, drowsiness, fainting, confusion) and cardio-respiratory syndromes (dyspnea, precordialgia). Systemic effects generally disappear rapidly with early first aid (hence, they are mostly described by the patients themselves) or may last for hours [16]. Serious envenomations have been reported from both sides of the Atlantic [39,40,41]. In 2011, about 10% of *Physalia physalis* victims in Aquitaine (France) presented life-threatening systemic conditions (especially respiratory distress), which required hospitalization [16]. Three fatal envenomations from *Physalia* have also been reported on the Southern Atlantic coasts of the United States [1,42].

Although first aid of *Physalia* stings is still quite controversial, the most effective treatment to alleviate pain is tentacle removal, followed by hot water application on the stung skin area. In a randomized controlled trial (RCT) conducted on 96 swimmers accidentally stung by *Physalia*, there was strong evidence (*p* < 0.002) of decreased pain at 10 and 20 min post-treatment with hot water immersion at 45 °C, as compared to ice packs [43]. These findings were somewhat confirmed by Bowra [44] in a cross-over study, which reported that victims treated with hot showers were more likely (*p* < 0.001) to benefit from stronger and quicker pain relief, as compared with those treated with ice packs.

Despite being conducted with a loose design (no statistical analysis and no controls employed), one study suggested ice packs as an effective remedy to alleviate pain from *Physalia* stings [45]. According to Exton [45], one single skin application of ice packs resolved pain in 100% of 82 victims with mild pain; a second ice pack application resolved the symptoms in 98% of another 45 patients suffering from moderate pain and in 75% of 32 patients suffering from severe pain.

Though being advocated by a few authors [7] and endorsed by laboratory evidence [46] and a RCT [47], topical vinegar is not universally accepted as treatment [1,31], as it seemingly increases nematocyst discharge from some *Physalia* species [1,48]. In particular, nematocyst discharge from the multi-tentacled *Physalia* species living in Australian waters was observed under the microscope after exposing segments of tentacles to methylated spirits (grade 5 in a scale 1 to 5) and acetic acid (grade 2) in the laboratory [49,50]. Furthermore, a recent *in vitro* study [51] confirmed that pieces of *Physalia physalis* tentacles exposed to vinegar promptly caused thousands of nematocysts to discharge. Nonetheless, there seems to be growing consensus toward vinegar use in *Physalia* stings, at least outside Australia, where the multi-tentacled species has not been reported thus far [1,52]. The likely mechanism of pain relief by vinegar is through blocking of additional nematocyst discharge [53].

Another effective remedy against pain from *Physalia* stings is Stingose (Hamilton Laboratories, Melbourne, Australia), an aqueous solution of 20% aluminum sulfate (MgSO_4_) and 1.1% surfactant. In particular, topical application of Stingose and various other substances were evaluated in a RCT, where the forearms of 20 volunteers were exposed to jellyfish tentacles [47]. Although subjective criteria for pain assessment were employed, the latter study evidenced that Stingose (similar to vinegar) was significantly more effective than sea water as a painkiller. Stingose, along with other chemicals, such as baking soda slurry, papain and bromelain, were beneficial against rupture of *P. physalis* nematocysts in the laboratory [46]. Weak evidence in favor of papain as effective treatment for *Physalia* stings was proposed [54]; however, this was subsequently questioned by Burnett [55]. Similarly to vinegar, lidocaine appeared to work as an analgesic, as well as a blocker of venom discharge from tentacles adhering to the skin [51].

By contrast, application of methylated spirits was associated with increased pain sensation in the RCT conducted by Turner [47]. Exposure to ammonia, ethanol and bromelain in the laboratory also reportedly increased nematocyst discharge from suspensions of *P. physalis* tentacles [51]. However, in the latter study, previous lidocaine application on jellyfish tentacle stings seemed to reduce pain sensation after subsequent exposure to ammonia, bromelain, ethanol and vinegar, as a result of presumed skin numbness, rather than inhibition of nematocyst discharge.

Reserpine was found beneficial in relieving spasm of the brachial artery following a probable second contact with a *Physalia* sp. in one case report [56], and anti-histamine i.v. resolved a dramatic collapse with cardio-respiratory arrest and mydriasis 15 min after a sting from *Physalia* sp. in another case report [57].

## 3.2. Cubozoans Species

Cubozoans, almost invisible in water, represent the most significant hazard among the jellyfish classes. Cubozoans are reportedly found swarming along coasts, in harbors and shallow waters, where they gather at different periods of time [58]. Two different orders of cubozoans are recognized: the large multi-tentacled chirodropids (among the most dangerous marine creatures) and the smaller four tentacled carybdeids [1,2,59].

### 3.2.1. *Chironex fleckeri*

*Chironex fleckeri*, named in honor of Hugo Flecker, a physician from Cairns (Queensland, Australia [60]), is responsible for several sudden and painful fatalities in Australian waters [1,2,61]. This dangerous box jellyfish can weigh up to 6 kg, with the diameter of its semitransparent cubic bell measuring about 20–30 cm. Four bundles of 10–15 translucent tentacles stem from four pedalia; the tentacles of mature specimens are generally flat and may extend up to 3 m [1].

*C. fleckeri* frequently moves from the open sea into shallow water to search for small prawns [1]. Therefore, shallow waters in proximity to beaches are the most frequent sites of stings [1].

*C. fleckeri* is considered an in-shore or coastal species, as it has never been found off-shore around coral reefs [1].

*C. fleckeri* is almost impossible to be seen by the victim, even after its sting [1,2]. Although the majority of stings from *C. fleckeri* are a minor event, with local pain and skin changes visible in the stung skin area, massive envenomation may cause severe systemic symptoms and sometimes death (usually within few minutes) [31].

Local signs of envenomation are intense skin pain with large (0.5–1.0 cm), edematous and erythematous plaques in the stung skin areas [31]. These wheals resemble whip marks. Blisters soon follow, leaving full-thickness areas of skin necrosis after the healing process (usually about 10 days). Permanent scars, which may have areas of abnormal pigmentation, are common residual patterns [1]. The severity of stings is proportional to the bell size, being extremely dangerous for *C. fleckeri*, with a diameter of 15 cm or more [1].

Systemic reactions to box jellyfish stings may include: dyspnea, hypotension, unconsciousness, arrhythmias and cardiopulmonary arrest [1,31]. *Chironex fleckeri* venom is also cardiotoxic, with a lethal component that is yet to be identified [1,62,63,64]. Experimental evidence suggests that *C. fleckeri* toxin can have a direct effect on the heart muscle and vascular tissue [65]. A total length of wheals of 6–7 m can cause quick loss of consciousness within seconds, with probable death even after 5–20 min or so following the envenomation [59,66]. However, severe envenomation and death is possible even with smaller skin contact [31]. Children seem to be more vulnerable to the effects of *C. fleckeri* venom, its lethality apparently being more likely in victims with a lower body mass [66]. Death usually occurs rapidly, as a result of cardiac asystole [2,12,31,64,66,67,68].

Presently, the appropriate first-aid management of suspected *C. fleckeri* stings is retrieval of the victim from the water and Basic Life Support (BLS) or Advanced Life Support (ALS), depending on the victim’s condition. Early application of vinegar (4%–6% acetic acid) for 30 s by dousing the stung skin area is the mainstay to prevent further discharge of venom from remaining nematocysts [1,31]. Adherent tentacles can be removed from the victim with the rescuer’s fingers, which will only incur relatively harmless prickling when handling them, due to the thick palmar skin (but this does not apply to other skin areas). Caution should however always be observed when handling live tentacles. It is advised not to waste time if vinegar is not available [1,2].

Laboratory studies suggest vinegar relieves pain by preventing further nematocyst discharge [53]. After vinegar application, minor stings respond well to ice pack application [64]. Opiates, such as fentanyl, are used for severe skin pain [31].

In a RCT, Stingose did not prove as effective as vinegar in inactivating nematocysts of *C. fleckeri* [69].

In their characterization of *C. fleckeri* toxin, Baxter *et al*. [27] showed that heat, formalin and ethylenediaminetetraacetic acid reduced all activities of the venom. The latter study hypothesized the potential of hot water immersion therapy for box jellyfish envenomation. Another experimental investigation supported the application of hot water at 43 °C or more, and *C. fleckeri* venom was found to lose lethality more rapidly the longer the exposure time. Lethality was assessed by time required to obtain ventricular arrest in crayfish injected with *C. fleckeri* tentacles treated with hot water [70]. However, the clinical application of heat on *C. fleckeri* venom appears to be impractical, as death after its stinging may occur within minutes [2]. Moreover, although hot water appears to be an effective treatment for pain [71], its use might be questionable, as the greatest hazard from *C. fleckeri* venom is a cardiotoxin [32,62]. Since the latter toxin can be carried directly and quickly into the circulatory system, applied heat may increase the blood flow, therefore resulting in increased severity of the cardiotoxic effects [72]. Carrette [70] appropriately concluded that if the sting is minor or unnoticed and systemic symptoms have already developed, heat application may be useless. In some cases, however, heat is included in the management of *C. fleckeri* envenomations [73]. A RCT is currently underway evaluating the effectiveness of heat in treating *C. fleckeri* envenomation (Seymour JE, personal communication).

In one case, the worsening clinical condition of a *C. fleckeri* victim following topical fresh water improved rapidly after alcohol application [12]. However, laboratory tests by Hartwick [53] reported massive nematocyst discharge following exposure of *C. fleckeri* tentacles to alcohol and methylated spirits.

*C. fleckeri* anti-venom obtained by hyper-immunizing sheep has been widely available since the 1970s and is produced by Commonwealth Serum Laboratory (Melbourne, Australia) [1,31,59,66]. Anti-venom ampoules of 20,000 units can be given intravenously over five minutes [59,66]. In order to control local symptoms (pain and local tissue damage), as well as more severe systemic effects, the anti-venom should be administered as early as possible. Although no significant adverse reactions to this antidote have been reported, there is a possibility of an allergic reaction. With consideration that the majority of victims envenomed by *C. fleckeri* have minimal symptoms, it is best to restrict anti-venom use to situations of cardio-respiratory instability, cardiac arrest, arrhythmias, difficulty in swallowing, severe pain resistant to opiates or significant risk of skin scarring [1,31,34]. Experiments in rats envenomed by *C. fleckeri* showed that a combination of the anti-venom and MgSO_4_ prevented cardiovascular collapse [74].

### 3.2.2. *Chiropsalmus quadrigatus*

*Chiropsalmus quadrigatus* is a very common species in the Indo-Pacific Ocean from Australia to the Philippines/Japan, being less frequent in the North American and Caribbean seas [2,3]. However, identification is difficult, and many different jellyfish in the Western Pacific Region are incorrectly classified as *C. quadrigatus* [75]. When searching for food (shrimps), this jellyfish species swims on the sea surface and can be frequently found in shallow coastal waters [1].

*C. quadrigatus* is smaller than *C. fleckeri*, the former having a bell of about 7–10 cm, with taxonomic expertise being required to distinguish *C. quadrigatus* from immature *C. fleckeri* [1]. *C. quadrigatus* exhibits from the four lower corners of its cubic bell one pedalium comprising 9–15 rounded tentacles about 3 m long [1].

Stings from this species cause immediate symptoms of intense pain, redness and swelling subsiding after a number of minutes, but persisting for about 24 h. Systemic symptoms (including allergic dermatitis, hypertension, bradycardia, cardiac asystole, respiratory failure with pulmonary edema, shock and thousands of deaths) have been reported along the Pacific coastal areas, but not in Australia [1,2,57,76]. *C. quadrigatus* envenomation is generally milder than *C. fleckeri*’s [47]. For this reason, it is practically impossible to distinguish *C. quadrigatus* from mild *C. fleckeri* stings, along with difficulty in distinguishing nematocysts from skin scrapings of *C. fleckeri* and *C. quadrigatus* [1]. Nematocysts of all cubozoans retrieved from skin scraping are generally difficult to identify, due to a lack of comparative characteristics [77].

Despite the various species improperly classified as *C. quadrigatus*, the medical approach to manage their stings does not vary across the chirodropids, as all seem to cause very similar, if not identical, symptoms [75]. 

As for *C. fleckeri*, vinegar is also used in the management of *C. quadrigatus* stings; analgesics, such as fentanyl or morphine, are used in more painful stings [1].

*C. fleckeri* anti-venom, administered to mice exposed to *C. quadrigatus* toxin, proved to counteract the dermoneurotic, lethal and hemolytic effects [78] and prevented the myotoxic and neurotoxic effects *in vitro* [79], but not the cardiovascular effects of the venom [80]. Despite the latter endorsing experimental evidence, however, clinical evidence for the use of *C. fleckeri* anti-venom in *C. quadrigatus* envenomations is still lacking [31]. 

Electrically stimulated *Chiropsalmus* sp. nematocysts underwent further discharge following pressure immobilization bandage (PIB) in vitro. This was confirmed by microscopy before and after application of 40 mm Hg PIB [81].

### 3.2.3. *Chiropsalmus quadrumanus*

This species is found in warmer waters of the Atlantic Ocean from North Carolina to Brazil [82]. Its transparent cubic bell may have a diameter of up to 14 cm. Seven to nine pale mauve tentacles arise from the 4 palmate pedalia and may extend up to 3–4 m [2,3].

Contact with the tentacles of this jellyfish species can produce skin lesions, sometimes requiring medical attention [80], and can even be potentially lethal to children [2].

The beneficial effect of lidocaine was tested by Birsa *et al*. [51] in a two-arm non-randomized controlled trial, where two of the authors were stung on their forearms by *C. quadrumanus* and *Chrysaora quinquecirrha*. Immediate pain relief was obtained with 10% and 15% lidocaine solutions, but it took approximately one minute to achieve the same results with 4%–5% concentrations. More diluted preparations (1%–3%) required an even longer time (10–20 min). Solutions of benzocaine and ethanol were also able to elicit some benefit for skin pain, but only after 10 min. By contrast, some chemicals, such as ammonia, alcohol and vinegar, exacerbated the pain sensation.

### 3.2.4. *Carukia barnesi* and Other Australian Carybdeids

*Carukia barnesi* can be found throughout all Australian waters, particularly in Queensland and Northern Territories [1,61,75]. In summer, it can inhabit shallow coastal waters, as well as the deep sea off the Greater Barrier Reef [1]. This small transparent carybdeid presents a cubic bell 2 cm wide and 2.5 cm long. Its four tentacles have a length varying from a few mm to 35 cm [2].

*C. barnesi* stings are usually mild or unnoticed, and the creature is hard to see. Following the sting, an oval erythematous area of 4–7 cm with a cluster of 2 mm surrounding vesicles may appear within 20 min or may not be noticed [1,2]. Thirty-sixty minutes or so after the sting, severe systemic symptoms may occur: the Irukandji syndrome—named after an Aboriginal tribe formerly populating the area north of Cairns (Queensland, Australia) [83]. This potentially deadly syndrome was proposed by Flecker in 1952, who described a pattern of characteristic symptoms following a reportedly minor sting in North Queensland [84]. However, a number of cases occurred before the responsible creature was captured in 1961 and named by Southcott, combining the words “carybdeids” and “Irukandji” to create the term “Carukia” and “barnesi”, commemorating Dr. Barnes [60,83,85]. Irukandji syndrome can consist of severe low back pain, muscle cramps in all four limbs, abdominal and chest pain, tremors, sweating, anxiety, restlessness, nausea, vomiting, headache and palpitations [1,83,85,86,87]. Since its original description, life-threatening hypertension (up to 300/150 mm Hg) [75], tachycardia, pulmonary edema and toxic global heart dilatation requiring intensive care unit (ICU) admission have been added as further complications of this syndrome [62,83,86]. Depending on the species responsible, many patients with Irukandji syndrome have pain without hypertension, while others have severe hypertension, even with effective pain control [88]. Deaths from Irukandji syndrome have been reported, due to intracerebral hemorrhage secondary to severe hypertension caused by the envenomation [89]. Although some authors suggest that cardiac dysfunction could be attributed to a myotoxin [34], according to others, it is only caused by the release of vast amounts of noradrenaline [90]. In particular, it has been suggested that the Irukandji syndrome is caused by venom disruption of the sodium ions, generating massive catecholamine release [90]. The Irukandji syndrome can be associated with the occurrence of a calcitonin gene-related peptide [91], endogenous catecholamines and stress cardiomyopathy [92,93]. Raised troponin levels have been reported [94,95].

The syndrome, initially described in Australia, certainly exists also in other areas, such as Thailand [96], French West Indies [97] and Florida [13], but it is probably underestimated and not medically reported.

As most Irukandji syndromes are severe, it is advisable to transport the victim to hospital in any case. The recommended first aid for pain relief in *C. barnesi* stings is ice packs or hot water, and prevention of further nematocyst discharge is application of vinegar [2,98], though no conclusive evidence in favor of vinegar exists [83,99]. In case of Accident and Emergency (A and E) admission, the mainstays are parenteral opiate analgesia [94], antihypertensive medication (5 mg phentolamine i.v. [88]) and 10% calcium gluconate i.v. for muscle spasms [100]. In 10 cases of Irukandji syndrome, victims required high dosages of opiates to relieve pain and seemed to benefit from magnesium sulfate (MgSO_4_) i.v., which also resolved the hypertension [100]. Although magnesium is known to work effectively as an antihypertensive by reducing vascular resistance, its analgesic properties still remain unexplained [83]. Sublingual glyceryl trinitrate has also been used as a first aid pre-hospital treatment for Irukandji syndrome to transiently lower blood pressure [88], but no evidence exists for instituting such therapy to relieve patient pain or improve the victim’s ultimate outcome (pulmonary oedema or myocardial insult). Severe cases may require ALS with intubation and inotropic pharmacological support (dopamine and adrenaline), usually due to cardiac failure [83]. There is no evidence that *C. fleckeri* anti-venom is effective to treat the Irukandji syndrome, and no other antidote currently exists [1,31]. Although pain and hypertension can be managed with common medications, there is an urgent need for an antidote to contrast the cardiac effects induced by the jellyfish toxin [83].

Among 116 patients admitted to A&E departments for Irukandji syndrome in one study, *C. barnesi* nematocysts were found in 39 of the 40 victims undergoing skin scrapings, while the remaining case had nematocysts of uncertain identity [94]. However, in a retrospective/prospective case series of 87 people observed from 1997 to 2007 at health services in Northern Australia for symptoms consistent with Irukandji syndrome, nematocysts of variable morphology were reported from the skin scrapings of 7 patients, suggesting different species as putative agents [95].

Other carybdeid species have been recently identified in Northern and Western Australian waters as associated with cases of Irukandji syndrome [83,101]. *Carybdea xaymacana*, *Carukia shinju*, *Malo maxima*, *Alatina mordens*, *Malo kingi* and *Morbakka fenneri* (a large carybdeid resembling the Western Atlantic jellyfish *Tamoya haplonema*) seemingly produce a severe syndrome similar to *Carukia barnesi*; another species, named *Gerongia rifkinae* (first described in 2005), seems to cause milder health effects [83]. The Australian multi-tentacled *Physalia* sp. seems also to be implicated with an Irukandji-like syndrome consisting of back pain, dyspnea and anxiety [2,50,83].

### 3.2.5. *Carybdea rastoni*

This small four tentacles carybdeid is very common in all Australian waters, as well as the Western Pacific. The bell is 3 cm wide and 5 cm long, with tentacles ranging from 5 up to 30 cm [1].

Stings normally produce four wheal marks 3–12 mm wide, with delayed and moderate pain (for 2 h), swelling and erythema (generally for 2–3 days). Pigmentary changes may persist for two weeks after the sting [1]. Stings from this carybdeid species do not cause the Irukandji syndrome [83].

First aid for *C. rastoni* stings relies on the same measures as for *C. fleckeri* [1]. There is laboratory evidence that vinegar inhibits the firing of nematocysts [99], whereas alcohol and methylated spirits should be avoided, as they cause massive discharge. Other substances found to inactivate nematocysts are Stingose and baking soda slurry [99]; however, the latter are less available and more expensive than vinegar. Ice packs and local analgesic ointments may be effective painkillers in more severe cases [2].

### 3.2.6. *Carybdea alata*

*Carybdea alata* (also known as *Alatina moseri* [83]) is a tropical carybdeid species very common in Hawaiian and Eastern Pacific waters [2,87]. The bell, twice higher than wide, has a diameter measuring up to 230 mm and presents a blunt flat head. The four tentacles are generally longer than the umbrella diameter. The bell is normally milky white, and the tentacles are pink [2].

Stings from *C. alata* are generally not lethal. Pain is normally moderate to severe and lasts about 2 h; typical skin marks are four wheals of about 10–20 cm in length and 3–12 mm in width; subsequent vesicles and pigmentary changes may persist for up to two weeks after the sting [1]. Cases of anaphylaxis or anaphylactoid syndrome have been described [87]. *C. alata* is included among the species other than *C. barnesi* potentially able to cause the Irukandji syndrome [83,87].

The evidence-based first aid for pain treatment of *C. alata* stings in Hawaiian and Eastern-Central Pacific regions is hot water immersion [87,102].

In a paired RCT, 25 volunteers stung on both arms with *C. alata* tentacles were either treated with 40–41 °C hot water immersion on one limb and vinegar or papain on the other. Pain sensation at 4–20 min, assessed by visual analog scale (VAS), was significantly lower in the limbs treated with hot water. Nonetheless, assessing pain level from two simultaneous stings could be a possible element of confusion for the volunteers; moreover, control chemicals were previously described as potentially stimulating nematocyst release [102].

The analgesic properties of hot water were also assessed on 133 swimmers accidentally stung by *C. alata* in Hawaii [23]. Although this study suffered from a lack of randomization, blinding, intention to treat analysis and some other methodology issues, the odds ratio for pain cessation was 5.2 (95% confidence interval: 1.3–22.8) after 5–10 min following application of hot water, as compared to placebo [23,52].

Hot water immersion at 45 °C also proved to be the only treatment able to relieve skin pain on the forearms of five volunteers deliberately stung with *Carybdea* spp. tentacles (two stings per arm). Although hot water provided effective pain relief at 4–10 min post-application, vinegar, ice and aluminum provided unsatisfactory (*i.e.*, incomplete and/or temporary) benefits [103].

In another retrospective quantitative analysis of medical records from 113 jellyfish victims in Hawaii, hot showers were more likely to relieve pain than parenteral analgesics or tranquilizers, particularly in the case of Irukandji-like syndrome [87].

In a follow-up study of 62 swimmers accidentally stung by *C. alata* in Hawaii, Thomas [104] observed no significant difference at VAS pain serial measurements in victims treated with papain (*N* = 14), freshwater (*N* = 19), seawater (*N* = 16) and aerosolized Stingose (*N* = 13). However, randomization was not feasible in this study, loss of follow up at 10 min was substantial and responsible jellyfish species were not properly identified [54].

### 3.2.7. *Morbakka*

This large carybdeid found in Queensland is similar to *Tamoya* sp. [2]. It has a transparent bell normally 10 cm high and 6 cm large [84]. Contact with its tentacles causes an immediate burning pain lasting approximately 24 h, with wheals 20 mm wide [2].

A number of substances have been tested in laboratory by Fenner *et al*. [105] on *Morbakka* tentacles with sea water as control. Vinegar fully inhibited nematocyst discharge, whereas methylated spirits caused 100% rupture. Other chemicals, such as Stingose, baking soda slurry and sodium hypochlorite, did not cause discharge from the tentacles determined by microscopic examination; however, if the same treated tentacles were subsequently applied onto the forearm, skin itching and erythema occurred [105].

## 3.3. Pelagiidae Species

Although distributed worldwide, Pelagiidae are more common in warm waters, especially the Mediterranean Sea (*Pelagia noctiluca*) and African and coastal Brazilian waters (*Chrysaora* spp.) [1,2,25,106]. These medusae are typically found in the open sea and frequently gather in swarms along bays and estuaries.

### 3.3.1. *Pelagia noctiluca*

*Pelagia noctiluca* is an ubiquitous species, being distributed in tropical, as well as Northern Atlantic and Pacific oceans [1]. It is reportedly very common in the Mediterranean Sea [25,107].

This medusa has a hemispherical bell whose diameter usually measures about 12 cm and presents numerous stinging wart-like protuberances. The bell can be transparent to fluorescent light pink, colored by violet warts. Sixteen marginal 30–40 cm long tentacles stick out from the umbrella, with mouth arms about five-times the bell height. The tentacles and the mouth arms are normally colorless or red/magenta [2,3].

*Pelagia noctiluca* is a severe stinging jellyfish causing local symptoms, such as pain, erythema, edema and vesicles. This jellyfish is able to restore its stinging capacity after only a few days following nematocyst liberation [25]. Although very painful, *P. noctiluca* stings are usually not considered life-threatening, as systemic symptoms are relatively uncommon [1,25]. Cases of anaphylaxis post-envenomation with *P. noctiluca* have been reported in the literature [108].

Sea water and ice packs appear to be the most appropriate management of stings from this species, whereas vinegar should be avoided, as it triggers nematocyst discharge [1,98,106,109].

Discharge of *P. noctiluca* nematocysts was stimulated in laboratory by anionic solutions, such as Cl^−^ and especially I^−^, whereas venom release was blocked by cations, such as Mg^2+^, as well as Ba^2+^ and, above all, Ca^2+^, which were also capable of contrasting the effect of iodide solutions on venom discharge [110].

### 3.3.2. *Chrysaora quinquecirrha*

Also called the “sea nettle”, this species is widely distributed in the Atlantic, Indian and Pacific Oceans [1]. *C. quinquecirrha* has a bell diameter of about 6 cm, sometimes up to 25 cm; the exumbrella is smooth, with the tentacles (generally 40 in adults) extending up to 3–4 m [1,2,3].

These medusae have a greatly varying coloration: colorless, transparent, whitish or yellowish, with mouth-arms of the same colors as the bell. Some also have variously developed radial streaks. Marginal tentacles can be colorless, yellow, red or of intermediate color [2,3].

Contact with *C. quinquecirrha* tentacles causes normally mild to moderate pain, and medical care may be necessary [2]. The skin rash consists of erythematous wheals and pink spots that may persist for up to one month [1,51].

Given its similarity to *Pelagia noctiluca*, *C. quinquecirrha* nematocysts seem also to be activated by vinegar [109]. As for *Physalia physalis*, nematocyst discharge was found after exposure of a suspension of *C. quinquecirrha* tentacles to acetic acid (5%), ethanol (70%), ammonia (20%) and bromelain (meat tenderizer), with ammonia having the strongest effect [51].

Ethanol-Benzocaine blends and especially high concentrated (10%–15%) preparations of lidocaine reduced pain and decreased skin redness post-*C. quinquecirrha* stings. By contrast, exposure to deionized water, meat tenderizer and urea did not produce any benefit [51], and ammonia, ethanol and vinegar increased pain sensation.

Arnold [54] provided weak evidence that papain was effective in treating pain from *C. quinquecirrha* stings. This result was confirmed by Burnett *et al.* [46], who performed laboratory tests to assess the effect of different substances on nematocyst discharge from *Chrysaora* tentacles. High concentrations of meat tenderizer and papain were effective along with Stingose, although rinsing the treated skin exacerbated pain sensation. Conversely, substances found to trigger nematocyst discharge in the latter study were sodium hypochlorite, sodium hydroxide, acetone, vinegar (5%) and ammonia. A very effective remedy to prevent nematocyst discharge was baking soda slurry, unless previous exposure to sodium hypochlorite had occurred. Baking soda is the suggested first line therapy to inhibit nematocyst discharge for *Chrysaora* stings [42,46].

## 3.4. Cyaneidae Species

This family is closely related to Pelagiidae. Members of the Cyaneidae are ubiquitous, being very common in warmer, as well as colder, waters. Unlike Pelagiidae, Cyaneidae are found in shallower waters, and their tentacles are formed in clusters. The largest *Cyanea* spp. are found in polar regions [2,3].

### Cyanea capillata

*Cyanea* *capillata* populate waters throughout the world, being found everywhere in Australia, as well as being the most common jellyfish along the Norwegian coast and the North Sea [2,111,112].

The bell of this medusa varies from pink to reddish-gold or brownish-violet, with purple oral arms and reddish or yellow tentacles, hence the common name “Lion’s Mane”. The umbrella ranges from 30 to 80 cm in diameter, in some cases even being almost 2 m [2,111,112].

Stings from *C. capillata* cause either minor or more severe skin pain/discomfort, and any swelling normally resolves after about 15 min, with erythematous stripes potentially remaining for several days [2].

Systemic symptoms, such as nausea, sweating, abdominal and muscular cramps, may sometimes occur soon after the sting [1]. Detached tentacles are still capable of envenomation.

Vinegar is contraindicated also for *C. capillata* [112]. Fenner and Fitzpatrick [109] tested *Cyanea capillata* nematocyst discharge after exposing its tentacles to different solutions of vinegar, methylated spirits and seawater. Vinegar caused discharge, whereas sea water and methylated spirits did not. There was no difference in the results whether the test was conducted on tentacles attached to the medusa or on isolated tentacles. Alcohol, acids and urea were also found to cause nematocyst discharge [109].

In his trial without controls and statistical analysis, Exton *et al.* [45] reported ice pack application to also be an effective painkiller for *C. capillata*. Similar to *Chrysaora*, baking soda is also recommended for *Cyanea* stings [42]. Therefore, after rinsing with sea water and removing the tentacles, ice application and baking soda is currently considered the primary first aid approach.

## 3.5. Pelagic Cnidaria: Distribution, Envenomation and Treatment

Table 1 shows the geographical distribution and envenomation effects of the main stinging pelagic Cnidaria.

**Table 1:** Geographical distribution and effects of venom of the main stinging pelagic Cnidaria.

Species and Size	Geographic Distribution	Local Symptoms	Systemic Symptoms; (Deadly = D)
*P. physalis* Float: 2–30 cm high Tentacles: 10–30 m	worldwide, more common in tropical waters [1,2,7,16]	acute pain, wheals ≥7 cm, skin necrosis after 24 h (++/+++) [1,2,16]	muscular spasms, abdominal pain, arrhythmias, headache, D [1,2,16]
*P. utriculus* Float: 2–10 cm high Single tentacle: 2–5 m	Tropical Indo-Pacific ocean, Australia, South-Atlantic [1,2]	local pain, wheals (+/++) [1,2]	very rare or none [1,2]
*C. fleckeri* Bell: (20 × 30) cm Tentacles: 2–3 m	Indo-Pacific region and Australia [1,2]	pain, massive wheals, vesicles for 10 days, scarring (+++) [1,2,31]	severe hypotension, cardiac failure/arrest, arrhythmias, pulmonary hypertension, D [1,31]
*C. quadrigatus Bell: (10 × 8) cm Tentacles: 5–30 cm*	Australia, Indo-Pacific region [1,2]	pain, wheals, swelling for 24 h (++/+++)	asystole, bradycardia, hypotension, pulmonary hypertension/oedema, D [1,59,75]
*C. quadrumanus Bell Φ: (14 × 10) cm Tentacles: 3–4 m*	North West Atlantic, Caribbean and Brazil [6,82]	pain, wheals (for 24 h); scarring and dyschromia for 2 months (++/+++) [2,82]	hypotension, acute cardiac failure, pulmonary hypertension/oedema, D [2,82]
*C. barnesi Bell Φ: (2.5 × 2) cm Tentacles: 5–35 cm*	Australia [1,2]	oval erythema 5 × 7 cm with surrounding papules (+/++) [1,2,83]	Irukandji syndrome (back pain, severe hypertension, agitation, muscle cramps, headache, nausea/vomiting, sweating), D [1,2,83]
*Morbakka Bell: (11 × 5) cm Tentacles: 10 cm*	Australia [1,2]	10 mm wide wheals, intense pain, itching, vesicles, skin necrosis (++/+++) [1,2,83]	Irukandji syndrome (muscle spasms, back pain, anxiety, respiratory distress, hypotension, sweating) [1,2,83]
*C. rastoni Bell: (5 × 2) cm Tentacles: 5–30 cm*	Australia [1,2]	delayed and moderate pain, wheals (3–12 mm width), swelling, blisters (rare), pigmentary changes for 2 weeks after sting (++) [1]	No [1]
*C. alata Bell: (9 × 5) cm Tentacles: 30–40 cm*	tropical and sub-tropical Pacific waters, Hawaii [2,75,87]	pain, wheals, blisters, dyschromia for 2 weeks (++/+++) [2,51,75,87]	mild Irukandji syndrome, possible allergic reactions [52,83,87]
*Tamoya haplonema Bell: (10 × 5) cm Tentacles: 3 cm*	Atlantic ocean (tropical/sub-tropical waters) [2,7,75]	burning pain (for about 2 h), wheals, blisters/scarring (++) [2,7,75]	muscle cramps, nausea, vomiting, restless, sweating, headache [2,7,75]
*P. noctiluca* Bell: (10 × 3) cm Tentacles: 10 m	worldwide, tropical and cold waters (common in Mediterranean, North Atlantic, North Pacific) [1,2]	instant severe pain, wheals, possible hyperpigmentation (++/+++) [1,2,106]	(rare) allergic reaction and respiratory distress [1,2,105]
*C. quinquecirrha* Bell Φ: 6 cm Tentacles: 50 cm	Australia, Atlantic, Pacific, Indian ocean [1,2]	intense pain, wheals/rash for days (+/++) [1,51]	(rare) allergic reaction and respiratory distress, D [1,51]
*C.* *capillata* Bell Φ: ≥1 m Tentacles: 30–50 cm	worldwide; more common in North Sea, North Atlantic, Arctic Sea, North Pacific [1,2,17]	Pain, wheals, erythema may persist for days (++/+++) [1,2,17]	(possible) muscle cramps, sweating, nausea, allergic reaction [1,2,17]

Φ = diameter of the jellyfish bell/umbrella. Intensity of local symptoms: + = mild; ++ = moderate; +++ = severe.

Table 2 and Table 3 present the evidence of treatment for jellyfish stings, according to the criteria established by the Agency for Healthcare Research and Quality (AHRQ) [113].

**Table 2:** Treatment guidelines and corresponding level of scientific evidence for *Cubozoans* spp. (references in superscript).

	Chirodropids		Carybdeids			
	*C. fleckeri*	*C. quadrumanus*	*C. alata*	*C. barnesi*	*C. rastoni*	*Morbakka*
Sea water rinsing	A,B(4) ^[1,2]^	B(4) ^[51]^, F(2) ^[51]^	A,B(1,4) ^[1,2,103,104]^	A(4) ^[1,2]^	A(4) ^[1,2]^	
Hot water/packs	A,B(4) ^[26]^, E(4) ^[70,71]^, F(4) ^[70]^		A(1,3) ^[23,52,83,87,103]^	A(4) ^[83]^		
Tentacle removal	B(4) ^[1,2]^	B(4) ^[1,2]^	B(4) ^[1,2,87]^	B(4) ^[1,2]^	B(4) ^[1,2]^	
Topical Vinegar	A,B(1,4) ^[1,51,69]^	A,B(4) ^[7]^, C(4) ^[51]^	C(4) ^[87]^, F(4) ^[103]^	B(4) ^[2]^	B(4) ^[99^]	B(4) ^[105]^
Ice packs	A(1) ^[64]^	D(2) ^[51]^	C(4) ^[87]^, F(4) ^[103]^			
Fresh water	C(4) ^[11,12]^	F(2) ^[51]^	A(1) ^[104]^, C(4) ^[12]^			
BaCl_2_						
MgCl_2_						
NaOH						B(4) ^[105]^
NaCl						
NaClO						
Choline-Cl						
MgCl_2_ solution						
Urea	C(4) ^[53]^	C(4) ^[51]^				
Stingose ^§^	A,B(1,4) ^[69]^		A(1) ^[104]^, F(4) ^[103]^		B(4) ^[99]^	B(4) ^[105]^
Acetone						
Bromelain 10%		C(4) ^[51]^, F(2) ^[51]^				
Papain			A(1) ^[104]^			
Baking soda slurry ^†^					B(4) ^[99]^	B(4) ^[105]^
Methylated spirits	C(4) ^[53]^				C(4) ^[99]^	C(4) ^[105]^
Ammonia		C(4) ^[51]^, D(2) ^[51]^				
Ethanol	A,E(4) ^[11]^, C(4) ^[53]^	C(4) ^[51]^, D(2) ^[51]^			C(4) ^[99]^	
Topic lidocaine		A(2) ^[51]^, B(4) ^[51]^				
Opiates i.v.	A(4) ^[1,31]^	A(4) ^[1,31]^		A(4) ^[83]^		
MgSO_4_^♥^ i.v.	E(4) ^[74]^			A,E(4) ^[100]^		
Reserpine i.v.						
Phentolamine i.v.				E(4) ^[83]^		
Glyceryl trinitrate ^♣^				E(4) ^[88]^		
Antihistamine i.v.						
Anti-venom	A,E(1) ^[59,78]^	E(4) ^[59,78]^ *	F(4) ^[59]^	F(4) ^[59]^	F(4) ^[59]^	F(4) ^[59]^
PIB	C(4) ^[114]^, E(4) ^[115]^	C(4) ^[81]^ *, E(4) ^[114]^				

A: pain relief; B: blocking of venom discharge; C: increased venom discharge; D: pain exacerbation; E: systemic benefits; F: ineffective; ^♥^ Magnesium sulfate; ^§^ 20% Al_2_(SO_4_)_3_; ^†^ NaHCO_3_; ^♣^ sublingual; * tested on *C. quadrigatus*; PIB: Pressure Immobilization Bandage; Level of scientific evidence according to the Agency for Healthcare Research and Quality [113]: 1 = Randomized controlled trials; 2 = Experimental paired/crossover study; 3 = Observational studies with controls; case series; 4 = Studies without controls, studies based on physiology and basic science, case reports and expert opinion.

**Table 3:** Treatment guidelines and corresponding level of scientific evidence for *Scyphozoans* and *Physalia* spp. (references in superscript).

	Physalia	Scyphozoans		
	*P. physalis*	*C. quinquecirrha*	*P. noctiluca*	*C. capillata*
Sea water rinsing	A(1) ^[1,2]^, B(4) ^[51]^	B(4) ^[46,48]^, F(2) ^[51]^	A(4) ^[109,110]^	A(4) ^[109]^
Hot water/packs	A(1,4) ^[43-45]^, F(4) ^[55]^	F(4) ^[55]^		
Tentacle removal	B(4) ^[1,2]^	B(4) ^[1,2,106]^	B(4) ^[1,2,110]^	B(4) ^[110]^
Topical Vinegar	A(1,4) ^[46,47]^, B(4) ^[46,48,55]^, C(4) ^[7,49]^	C(4) ^[46]^, D(4,2) ^[51]^	C(4) ^[109]^	C(4) ^[106]^
Ice packs	A(1,4) ^[43-45]^	F(4) ^[46,48]^		A(4) ^[45]^
Fresh water	B(4) ^[49]^	F(2) ^[51]^		
BaCl_2_	B(4) ^[110]^		B(4) ^[110]^	
MgCl_2_	B(4) ^[110]^		B(4) ^[110]^	
NaOH	C(4) ^[46]^			
NaCl			C(4) ^[110]^	
NaClO	C(4) ^[46]^	C(4) ^[46]^		
Choline-Cl			C(4) ^[110]^	
MgCl_2_ solution	B(4) ^[110]^	C(4) ^[46]^		
Urea		F(4) ^[51]^		
Stingose ^§^	A(1) ^[47]^, B(4) ^[46]^	B(4) ^[46]^		
Acetone	C(4) ^[46]^	C(4) ^[46]^		
Bromelain 10%	A(1) ^[47]^, C(4) ^[51]^	C(4) ^[46]^, F(2) ^[51]^		
Papain	A(1,4) ^[54]^, F(4) ^[55]^	A(4) ^[46]^, B(4) ^[46]^		
Baking soda slurry ^†^	B(1) ^[47]^,C(4) ^[46]^	B(4) ^[46,109]^	B(4) ^[46]^	A(4) ^[42,110]^
Methylated spirits	C(4) ^[49]^, D(1) ^[47]^		B(4) ^[109]^	A,B(4) ^[109]^
Ammonia	C(4) ^[46,51]^	C(4) ^[51]^, D(4,2) ^[51]^		
Ethanol	C(4) ^[51]^	C(4) ^[51]^, D(2) ^[51]^	C(4) ^[109]^	
Topic lidocaine	A,B(4) ^[51]^	A(4,2) ^[51]^, B(4) ^[51]^		
Opiates i.v.	A(4) ^[31]^			
MgSO_4_^♥^ i.v.				
Reserpine i.v.	E(4) ^[56]^			
Phentolamine i.v.				
Glyceryl trinitrate ^♣^				
Antihistamine i.v.	E(4) ^[57]^	E(4) ^[108]^	E(4) ^[108]^	E(4) ^[108]^
Anti-venom	F(4) ^[78]^	F(4) ^[78]^		
PIB				

A: pain relief; B: blocking of venom discharge; C: increased venom discharge; D: pain exacerbation; E: systemic benefits; F: ineffective; ^♥^ Magnesium sulfate; ^§^ 20% Al_2_(SO_4_)_3_; ^†^ NaHCO_3_; ^♣^ sublingual; PIB: Pressure Immobilization Bandage; Level of scientific evidence according to the Agency for Healthcare Research and Quality [113]: 1 = Randomized controlled trials; 2 = Experimental paired/crossover study; 3 = Observational studies with controls; case series; 4 = Studies without controls, studies based on physiology and basic science, case reports and expert opinion.

## 4. Discussion

Treatment of jellyfish envenomation is primarily directed at [52]:

1. Alleviating the local effects of venom (pain and tissue damage);
2. Preventing further discharge of nematocysts;
3. Controlling systemic reactions, including shock.

The most important step after envenomation is basic life support (ABCs), with the aim of maintaining respiration and blood circulation [2,98], and tentacle removal, since as long as tentacles adhere to the skin, nematocysts continue to discharge venom [2,100]. Tentacle removal should, however, be delayed until the patient is stable [41].

It is still debated which is the most appropriate method for tentacle removal, as the procedures may stimulate further nematocyst discharge [52]. Flushing the stung skin area with sea water is recommended [2,44,50,100], as well as the use of tweezers to remove tentacles [112]. Tentacles may also be removed with bare hands, but it is best to immediately rinse off the rescuer’s fingers carefully afterwards to prevent secondary stings [42].

As toxins vary among jellyfish species [1,2,116], different remedies are necessary to control pain, additional venom liberation and local reactions. The ideal treatment would be readily available, cheap, effective for inactivating toxins of various jellyfish species and would prevent further discharge of venom [52]. Although there are differences between species, there seems to be evidence and consensus on oral/topical analgesics, baking soda, hot water, ice packs and (for cubozoans and non-Australian *Physalia*) topical vinegar. In tropical Australia, where the risk of life-threatening cubozoans is substantial, the Australian Resuscitation Council (ARC) recommends vinegar application followed by tentacle removal and ice pack application if the jellyfish responsible cannot be clearly identified as harmless. Sea water should be used if vinegar is not available. Out of the tropics, where non-life-threatening jellyfish species predominate, the priority is pain relief, and the first aid approach should be sea water rinsing followed by either hot water (42 °C for 20 min) for confirmed *Physalia* stings or ice packs for stings of unknown origin [98]. All measures that could cause massive discharge of nematocysts should be carefully avoided.

Ice seems to slow the diffusion of the venom, thus acting as a painkiller, whereas the mechanism of pain relief due to heat is still debated. It is unlikely that it could be attributed to the denaturation of the venom. Cnidarians’ toxins denature rapidly above 50 °C [117], but this condition probably does not occur on human skin, where temperatures would be lower and the inactivation time longer. Cnidarians’ venom may have already circulated away from the sting site at the time of initiating treatment. Furthermore, denaturing the venom is unlikely to affect the pain in an already existing injury. Some authors argue that heat may modulate the pain receptors, thus leading to a reduction in pain sensation [118].

High dosages of intravenous ascorbate were reported to decrease the pain 10 min after administration to a male stung in both legs by a medusa while he was net fishing at a Malaysian beach. The responsible jellyfish was not identified, but since the local symptoms initially increased after application of vinegar, it seems plausible that the species involved could have been a scyphozoan. Vitamin C is considered effective against various toxins [119].

PIB for jellyfish envenomation remains controversial and is considered potentially dangerous [114], with some Northern Australian territories not recommending it as first aid treatment [120]. A significant amount of venom might remain in not yet discharged nematocysts adhered to the patient’s skin, and PIB pressure may stimulate venom extrusion from them [81,114]. Moreover, PIB may even interfere with the ability of vinegar to prevent additional discharge of venom from unexploded nematocysts [114]. Some experts argue that the likely increased liberation of venom generated by PIB could be offset by the containment of the venom in the limbs and decreased systemic absorption of toxin due to bandaging [115]. This compartmentalization of the venom within the sting area in *C. fleckeri* stings appears limited, however, as the health effects of the toxin develop within minutes, suggesting involvement of the circulatory system rather than the lymphatics [114]. Although massive and fatal *C. fleckeri* envenomation might have intravascular venom injection from venom covered tubules penetrating blood vessels, as well as slower lymphatic drainage, the benefit of PIB in counteracting the movement of venom from the sting site via the lymphatics and small blood vessels has still to be further tested [1]. Furthermore, the only real benefit of PIB seems to be immobilization and subsequent decrease in blood and venom flow; however, this can also be accomplished by other means without compression bandages [114]. Thus, despite being advocated by experts and authorities [115,121], the ARC does not recommend the use of PIB [98,114].

Large trials on topical inhibitors as effective measures to prevent jellyfish stings are ongoing and look promising [21]. A skin inhibitor cream (Safe Sea^®^, by Nidaria Technology, Zemah, Jordan Valley, Israel) is already commercially available in many countries worldwide to provide practical and cheap protection for swimmers against jellyfish stings. This preparation was recently formulated as a waterproof sunscreen containing octyl methoxycinnamate and zinc oxide, allowing both jellyfish inactivation and sunburn protection with a single application. Safe Sea^®^ is effective in preventing nematocysts from firing, but is not helpful after the stinger has fired [21]. This product was applied on the forearm (and conventional sunscreen on the other) of healthy volunteers exposed to tentacles of *C.* *quadrumanus* and *C.* *fluorescen*s in one RCT and to tentacles of *C*. *capillata* in another more recent Norwegian RCT. Safe Sea^®^ was also tested in a field RCT conducted in Florida and Belize (areas where *C*. *quinquecirrha*, *C*. *quadrumanus*, *Lunuche ungulate* and *Physalia* are common). In all the above trials, Safe Sea^®^ did not eliminate the stings, but significantly reduced their frequency and severity [21,122,123]. Safe Sea^®^ is likely to be effective against a vast range of jellyfish species [21]. It would therefore be interesting to test it also with more dangerous species, such as *C*. *fleckeri*, *C*. *barnes*i and *P*. *physalis*.

In terms of preventive medicine, divers and swimmers in risky areas should wear personal protective equipment (PPE). PPE is, in fact, almost totally effective against all jellyfish stings and is routinely recommended in Australia for all people (tourists, locals and recreational divers) [24]. Full-Body Lycra^®^ suits have been used by divers since the early 1980s and seem to be the best choice for routine-use protective clothing against *C. barnesi* [24]. However, the recreational public when swimming in risky areas seems reluctant to use PPE, as it was estimated that less than 5% of Queensland beach users wear any type of stinger protection [24]. Those who cannot refrain from diving/swimming in risky areas due to their occupation should wear protective gear. As these suits often leave the face, hands and feet exposed, snorkelers and pearl divers in Western Australia modify them to cover the whole body and avoid severe and/or life-threatening jellyfish stings [21,124].

Stinger nets are also used in Australia to keep jellyfish out of highly used swimming areas; however, these barriers (which normally have 2.5 cm holes) are more likely to be effective against larger species, such as *C. fleckeri*, as smaller jellyfish like *C. barnesi* are able to get through the nets [21,24,125].

Adequate signage should be placed at beaches to notify tourists about the jellyfish risk; water should not be entered during risky months. Tropical species, such as the chirodropids, occur predominantly (but not exclusively) during the summer months in the boreal and austral hemispheres, being found almost year round at the equator [2,61,75].

The vulnerable population (especially tourists and children) should be targeted through health education on how to avoid envenomations, how to behave in the event jellyfish are seen in the water (although most chirodropids are never seen and for them PPE is essential) and in the event of stings (first aid guidelines). Although sometimes impractical, free ice packs, as well as vinegar, should be made available at distribution points. Vinegar is very cheap, simple to use and does not have an expiration date; hence, it is also easy to keep on a boat, in the trunk of a car or at the beach. Bathers should be instructed on first aid measures.

Epidemiological data on jellyfish risk should be routinely collected to better inform and educate those categories at risk on the appropriate treatment modalities. Published literature on stings has been both limited and conflicting, treatment guidelines still lack consensus and it is often difficult to identify the jellyfish species involved (especially in the case of cubozoans) [2,126].

## 5. Conclusions

Efficacy of treatment relies mostly on studies without controls, studies based on physiology and basic science, case reports and expert opinions. To date, only eight RCTs [21,23,43,47,102,104,122,123] and one non-randomized controlled trial [51] investigating jellyfish treatment/prevention have employed adequate controls. Further research is needed to develop a recognized protocol for the management of jellyfish stings, although observing a rigorous and sound methodology seems problematic (difficulties in recruiting patients, randomization, outcome assessment, *etc.*). It is essential in future trials to exactly identify the jellyfishes under study, as many different species may be present even in the same waters: the larger the study, the more varieties may be involved.

Despite the above limitations, there seems to be evidence and consensus on the efficacy of oral/topical analgesics, baking soda, hot water, ice packs and (only for cubozoans and non-Australian *Physalia*) topical vinegar. A skin inhibitor cream is effective in preventing nematocysts from firing, significantly reducing the frequency and severity of stings.

Dissemination of appropriate treatment modalities should be deployed to better inform and educate those at risk. Adequate signage should be placed at beaches to notify tourists of the jellyfish risk. Swimmers in risky areas should wear protective equipment.

## Supplementary Files

Supplementary File 1 Supplementary Information (PDF, 126 KB)
