# Supplementary material for: Jellyfish Stings and Their Management: A Review
Source: Mar Drugs. 2013 Feb 22;11(2):523–50. doi: 10.3390/md11020523 (PMC3640396; doi:10.3390/md11020523)
Supplement: Supplementary File 1 — Supplementary Information (PDF, 126 KB) [file marinedrugs-11-00523-s001.pdf]

Supplementary Information

Table S1. Main jellyfish species, geographical distribution/habitat and effects of venom.

Species and Size	Geographic Distribution	Local Symptoms	Systemic Symptoms	Deadly
P. physalis<br>Float: 2–30 cm high<br>Tentacles: 10–30 m	worldwide;<br>more common in tropical waters	acute pain, wheals ≥7 cm, skin necrosis after 24 h (++/+++)	muscular spasms, abdominal pain, arrhythmias, headache	Yes
P. utriculus<br>Float: 2–10 cm high<br>Single tentacle: 2–3 m	tropical Indo-Pacific Ocean, Australia, South-Atlantic	local pain, wheals (+/++)	Very rare or none	No
C. fleckeri<br>Bell: (30 × 20) cm<br>Tentacles: 3–60 m	Indo-Pacific region and Australia	pain, massive wheals, vesicles for 10 days, scarring (+++)	severe hypotension, arrhythmias, cardiac failure, pulmonary hypertension	Yes
C. quadrigatus<br>Bell: (10 × 8) cm<br>Tentacles: 5–30 cm	Australia, Indo-Pacific region	pain, wheals, swelling for 24 h (++/+++)	asystole, bradycardia, hypotension, pulmonary hypertension/oedema	Yes
C. quadrumanus<br>Bell: (14 × 10) cm<br>Tentacles: 3–4 m	North West Atlantic, Caribbean and Brazil	pain, wheals (for 24 h); scarring and dyschromia for 2 months (++/+++)	hypotension, acute cardiac failure, pulmonary hypertension/oedema	Yes
C. barnesi<br>Bell: (2.5 × 2) cm<br>Tentacles: 5–35 cm	Australia	oval erythema 5 × 7 cm with surrounding papules (+/++)	Irukandji syndrome (back pain, severe hypertension, agitation, muscle cramps, headache, nausea/vomit, sweating)	Yes
Morbakka<br>Bell: (11 × 5) cm<br>Tentacles: 10 cm	Australia	10 mm wide wheals, intense pain, itching, vesicles, skin necrosis (++/+++)	Irukandji syndrome (muscle spasms, back pain, anxiety, respiratory distress, hypotension, sweating)	No
C. rastoni<br>Bell: (5 × 2) cm<br>Tentacles: 5–30 cm	Australia	delayed and moderate pain, wheals (3–12 mm width), swelling, blisters (rare), pigmentary changes for 2 weeks after sting (++)	No	No
C. alata<br>Bell: (9 × 5) cm<br>Tentacles: 30–40 cm	tropical and sub–tropical Pacific waters, Hawaii	pain, wheals, blisters,<br>dyschromia for 2 weeks (++/+++)	mild Irukandji syndrome, possible allergic reactions	No

Table S1. *Cont.*

Tamoya haplonema<br>Bell: (10 × 5) cm<br>Tentacles: 3 cm	Atlantic ocean (tropical/sub–tropical waters)	burning pain (for about 2 h), wheals, blisters/scarring (++)	muscle cramps, nausea, vomiting, restlessness, sweating, headache	No
P. noctiluca<br>Bell: (10 × 3) cm<br>Tentacles: 10 m	worldwide, tropical and cold waters (common in Mediterranean, North Atlantic, North Pacific)	instant severe pain, wheals, possible hyperpigmentation (++)	(rare) allergic reaction and respiratory distress	No
C. quinquecirrha<br>Bell Φ: 6 cm<br>Tentacles: 50 cm	Australia, Atlantic, Pacific, Indian ocean	intense pain ,wheals/rash for days (+/++)	(rare) allergic reaction and respiratory distress	Yes
C. capillata<br>Bell Φ: ≥1 m<br>Tentacles: 30–50 cm	worldwide; more common in North Sea, North Atlantic, Arctic Sea, North Pacific	pain, wheals; erythema may persist for days (++)	(possible) muscle cramps, sweating, nausea, allergic reaction	No

Φ = diameter of the jellyfish bell/umbrella. Intensity of local symptoms: + = mild; ++ = moderate; +++ = severe.

Table S2. Guidelines for the management of jellyfish stings: Treatments and level of scientific evidence.

**Treatments**	**Chirodropids**		**Carybdeids**		**Physalia**	**True Jellyfish**		
	C. fleckeri	***C. quadrumanus***	***C. alata***	***C. barnesi***	***P. physalis***	***C. quinquecirrha***	***P. noctiluca***	***C. capillata***
Sea water rinsing	A(4)	A(4,2), B(4)	A(1)		A(1)	B(4), F(2)	A(4)	A(4)
Hot water/packs	A,B(4)		A(1,3)	A(4)	A(1,4)	F(4)		
Tentacles removal	B(4)	B(4)	B(4)	B(4)	B(4)	B(4)	B(4)	B(4)
Topic Vinegar	A,B(1), C(4)	A,B(4)	C(4)	C(4), F(4)	A(1), B(4), C(4)	C(4), D(4,2)	C(4)	C(4)
Ice packs	A(1)	C(4), D(2)	C(4)		A(1,4), F(1)	F(4)		
Fresh water	C(4)	C(4), F(2)	A(1), C(4)		C(4)	C(4), F(2)		
$BaCl_2$					B(4)		B(4)	
$MgCl_2$					B(4)		B(4)	
NaOH	C(4)				C(4)			
NaCl							C(4)	
NaClO	C(4)				C(4)	C(4)		

Table S2. *Cont.*

Choline-Cl							B(4)	C(4)
$MgCl_2$ solution					C(4)	C(4)		
Urea		C(4)				B(4)		
Stingose §	A,B(1,4)		A(1), C(4)		A(1), B(4), F(4)	B(4)		
Acetone						C(4)		
Bromelain 10%		C(4), F(2)			C(4), F(2)	C(4), F(2)	C(4)	
Papain			A(1)		A(1,4)	A(4), B(4)		
Baking soda slurr †					C(4)	A(4)	A(4)	A(4)
Methlated spirits	C(4)		C(4)		A(1), C(4)	C(4)	C(4)	A,B(4)
Ammonia		C(4), D(2)	A,B(4)			C(4), D(4,2)	C(4), D(4,2)	
Ethanol	A,E(4), C(4)	C(4), D(2)				C(4), A(2)	C(4), A(2)	
Topic lidocaine		A(2), B(4)			A,B(4)	A(4,2), B(4)	A(4,2), B(4)	
Opiates i.v.	A(4)	A(4)	A(4)	A(4)	A(4)			
$MgSO_4$® i.v.	E(4)			A(4), E(4)				
Reseprine i.v.					E(4)			
Phentolamn i.v.				E(4)				
Glyceryl trinitrate ♣				E(4)				
Anti–histamine i.v.					E(4)	E(4)	E(4)	E(4)
Ascorbate (i.v./oral)	A(4) @	A(4) @	A(4) @	A(4) @	A(4) @	A(4) @	A(4) @	A(4) @
Anti–venom	A,E(1)	E(4) ♥	Ineffective	Ineffective	Ineffective	Ineffective	Ineffective	Ineffective
PIB	C(4), E(4)	C(4), E(4)	C(4), E(4)		C(4), E(4)	C(4)	C(4), E(4)	

Table S2. *Cont.*

**Legend**	
A = pain relief	1 to 4 = level of scientific evidence according to the Agency for Healthcare Research and Quality [2002]
B = block of venom discharge	1 = randomized controlled trials
C = increase of venom discharge	2 = experimental paired/crossover study
D = pain exacerbation	3 = observational studies with controls; case series
E = systemic benefits	4= studies without controls, studies based on physiology and basic science, case reports and expert opinion
F = ineffective	[@] = one single case report on an unspecified jellyfish species
[®] Magnesium sulfate	
[§] 20% $Al_2(SO_4)_3$	
[†] $NaHCO_3$	
[♣] = sublingual	
[♥] = tested on *Q. quadrigatus* (a similar cubozoan)	
PIB = Pressure Immobilization Bandage	
